# Supplementary material for: Dietary Habits and Nutritional Status in Ecuatorian Children Aged 1–11 Years: A Systematic Review Highlighting the Dual Burden of Malnutrition
Source: Nutrients. 2025 Nov 19;17(22):3608. doi: 10.3390/nu17223608 (PMC12655085; doi:10.3390/nu17223608)
Supplement: Supplementary file 1 [file nutrients-17-03608-s001.zip › nutrients-3923133-supplementary.pdf]

Supplementary Materials

Table S1: Quality Assessment of Included Studies Using the CASPe Tool

This table presents the detailed quality assessment of each study included in the systematic review, conducted using the Critical Appraisal Skills Programme (CASPe) for cohort studies. Each study was scored on five key questions, with a total score out of 5, and then classified as High, Moderate, or Low quality based on the total score.

Author/Year	Title	Q1: Clear Objectives? (1-0.5-0)	Q2: Adequate Methodology? (1-0.5-0)	Q3: Clear Context/Participants? (1-0.5-0)	Q4: Minimized Bias Risk? (1-0.5-0)	Q5: Clear/Applicable Results? (1-0.5-0)	Total Score	Study Quality
**Pazmiño, Heredia & Yánez (2019)**	Risk factors related to nutritional deficiency in children from a central Andean community in Ecuador	1	1	1	1	1	5	High
**Pacheco et al. (2023)**	Relationship between nutritional status and cardiometabolic risk in schoolchildren	1	1	1	0,5	1	4,5	High
**Darroman et al. (2023)**	Determining nutritional status in children and older adults in peri-urban neighborhoods	1	1	0,5	1	1	4,5	High
**Gómez & Crespo (2022)**	Nutritional status and its relationship with eating habits in children aged 1 to 3 years attending the child development center	1	1	0,5	1	1	4,5	High
**Ocaña & Sagñay (2020)**	Malnutrition and its relationship with cognitive development in early childhood children	0,5	0,5	0,5	0,5	0,5	2,5	Moderate
**Díaz & Da Costa (2019)**	Characterization of eating habits and nutritional status of preschoolers	1	1	1	1	1	5	High
**Peralta et al. (2023)**	Academic performance and its relationship with nutritional status. Schoolchildren, Sayausí Millennium Educational Unit, Cuenca, Ecuador	1	1	1	0,5	1	4,5	High

**León et al. (2020)**	Validity and reliability of the instrument to determine factors that influence the nutritional status by excess in children from 5 to 9 years old	1	1	0,5	1	0,5	4	High
**Guanoluisa et al. (2022)**	Assessment of nutritional status in children and adolescents in the Quevedo canton	1	1	0,5	1	1	4,5	High
**Caizaluisa, Quishpi & Pucha (2024)**	Eating habits and nutritional status of school-aged children (5-11 years) according to the National Health and Nutrition Survey (ENSANUT) 2018	1	1	0,5	1	1	4,5	High
**Sánchez et al. (2020)**	Assessment of nutritional status in the school population aged 8 to 11 in the urban area of Milagro	1	1	0,5	1	0,5	4	High
**Escandón, Bravo & Castillo (2019)**	Nutritional status of children at the private early childhood education center-Azogues, 2019	1	1	1	1	0,5	4,5	High
**Méndez et al. (2023)**	Dietary intake and nutritional status in children aged 1 to 5 years from the indigenous community of Angochagua, Ecuador	1	1	0,5	0,5	1	4	High
**Hidalgo (2021)**	Relationship between maternal education and nutritional status of children aged 0 to 3 years in CDIs in Salasaka, Tungurahua, 2016	1	1	0,5	0,5	1	4	High
**Vera, Zambrano & Ronquillo (2023)**	Eating habits in children aged 4 to 6 years at an educational institution in the province of Santa Elena, Ecuador	1	1	0,5	1	1	4,5	High
**Álvarez et al. (2020)**	Current perception of eating habits and nutritional status in children from the "Darío C. Guevara" Educational Unit in the parish of El Salto, Babahoyo - Los Ríos	1	1	1	1	1	5	High
**Pozo & Vargas (2022)**	Eating habits and their relationship with nutritional status and	1	1	1	0,5	1	4,5	High

	dyslipidemia in children aged 6 to 11 years
